# FGF-23 and sclerostin in serum and bone of CKD patients 

**DOI:** 10.5414/CN111111

**Published:** 2023-03-27

**Authors:** Florence Lima, Marie-Claude Monier-Faugere, Hanna Mawad, Valentin David, Hartmut H. Malluche

**Affiliations:** 1 Division of Nephrology, Bone and Mineral Metabolism, University of Kentucky, Lexington, KY, and; 2 Medicine-Nephrology and Center for Translational Metabolism and Health, Northwestern University, IL, USA

**Keywords:** FGF-23, sclerostin, bone, bone histomorphometry, CKD, renal osteodystrophy

## Abstract

Aims: Renal osteodystrophy occurs in the early stages of chronic kidney disease (CKD) and progresses during loss of kidney function. Fibroblast growth factor (FGF)-23 and sclerostin, both produced by osteocytes, are increased in blood of patients with CKD. The aim of this study was to analyze the impact of decline in kidney function on FGF-23 and sclerostin protein expression in bone and to study their relationship with their serum levels and bone histomorphometry. Materials and methods: 108 patients aged 25 – 81 years (mean ± SD: 56 ± 13 years) underwent anterior iliac crest biopsies after double-tetracycline labeling. Eleven patients were CKD-2, 16 were CKD-3, 9 were CKD-4 – 5, and 64 CKD-5D. Patients were on hemodialysis for 49 ± 117 months. 18 age-matched patients without CKD were included as controls. Immunostaining was performed on undecalcified bone sections to quantify FGF-23 and sclerostin expression. Bone sections were also evaluated by histomorphometry for bone turnover, mineralization, and volume. Results: FGF-23 expression in bone correlated positively with CKD stages (p < 0.001) increasing from 5.3- to 7.1-fold starting at CKD-2. No difference in FGF-23 expression was seen between trabecular and cortical bone. Sclerostin expression in bone correlated positively with CKD stages (p < 0.001) with an increase from 3.8- to 5.1-fold starting at CKD-2. This increase was progressive and significantly greater in cortical than cancellous bone. FGF-23 and sclerostin in blood and bone were strongly associated with bone turnover parameters. Expression of FGF-23 in cortical bone correlated positively with activation frequency (Ac.f) and bone formation rate (BFR/BS) (p < 0.05), while sclerostin correlated negatively with Ac.f, BFR/BS, and osteoblast and osteoclast numbers (p < 0.05). FGF-23 trabecular and cortical expressions correlated positively with cortical thickness (p < 0.001). Sclerostin bone expression correlated negatively with parameters of trabecular thickness and osteoid surface (p < 0.05). Conclusion: These data show a progressive increase in FGF-23 and sclerostin in blood and bone associated with decrease in kidney function. The observed relationships between bone turnover and sclerostin or FGF-23 should be considered when treatment modalities are developed for management of turnover abnormalities in CKD patients.

## Introduction 

The chronic kidney disease mineral and bone disorder (CKD-MBD) and its bone manifestation renal osteodystrophy is seen in virtually all patients requiring replacement of kidney function by dialysis. Renal osteodystrophy is characterized by abnormalities in bone turnover, mineralization, and volume [1, 2]. Increased blood levels of fibroblast growth factor (FGF)-23 are found early during the development of CKD-MBD and are considered a compensatory mechanism to avoid hyperphosphatemia and resultant hyperparathyroidism and turnover abnormalities [3, 4, 5]. There is also evidence that very high FGF-23 blood concentrations are associated with less mineralization abnormalities [6, 7]. Increased FGF-23 blood levels are also associated with significant progression of loss of kidney function [8] and increased mortality independent of serum phosphorus levels [9]. High FGF-23 blood levels have been found to be correlated positively with dialysis vintage and parathyroid hormone (PTH) [6] and were found to be associated with cardiovascular disease in hemodialysis patients [10, 11]. 

Sclerostin concentrations in blood have also been found to be increased in early stages of CKD [12, 13, 14]. Sclerostin is expressed in osteocytes and is an inhibitor of osteoblast activity resulting in lower bone formation, uncoupling between osteoblasts and osteoclasts, and lower bone volume [15]. Reduction of kidney function in sclerostin knockout mice results in development of renal osteodystrophy-typical bone abnormalities without a negative effect on bone mass [16]. Serum sclerostin was also found to be an independent predictor of mortality in dialysis patients [17]. 

There is limited information on bone protein expression of FGF-23 and sclerostin. The aims of this study were to evaluate: 1) bone expression of FGF-23 and sclerostin; and 2) their relationships with histologically determined bone turnover parameters and serum levels in patients with various degrees of CKD. 

## Materials and methods 

### Patients 

Consecutive patients with varying severity of renal failure enrolled in the Bone Registry at the Bone Diagnostic and Research Laboratory of the University of Kentucky were screened for participation in the study. The study was reviewed and approved by the Institutional Review Board at the University of Kentucky and was conducted according to the Declaration of Helsinki. All patients signed informed consent. Age-matched adult subjects with no known kidney disease were included as controls. 

Inclusion criteria were: patients aged ≥ 18 years, reduced kidney function documented by estimated glomerular filtration rate (eGFR) levels < 90 mL/min, no or steady dose of vitamin D or vitamin D analogs for ≥ 6 months. Exclusion criteria were previous renal transplantation or parathyroidectomy, use of calcimimetics, and medications known to affect bone metabolism such as glucocorticoids (except vitamin D), and life-threatening comorbid conditions such as HIV, malignancy, active infectious disease, cardiac and hepatic abnormalities. All patients underwent anterior iliac crest bone biopsies after tetracycline double labeling for mineralized bone histology and histomorphometry. Patients were given oral declomycin 300 mg twice daily for 2 days followed by a 10-day tetracycline-free interval and another course of tetracycline hydrochloride at 250 mg twice daily for 4 days. Iliac crest bone biopsies were performed after an additional 4 days without tetracycline administration. 

### Serum biochemistry 

Blood was drawn at the time of biopsy for determinations of serum levels of calcium, phosphorus, bone-specific alkaline phosphatase (BSAP), tartrate-resistant acid phosphatase 5b (TRAP5b), intact PTH, FGF-23, and sclerostin. Serum calcium and phosphorus levels were measured by automated techniques. Intact PTH was measured by a radioimmunometric assay (Scantibodies, Santee, CA, USA), normal range: 14 – 66 pg/mL; intra-assay coefficient of variation: < 5%. BSAP and TRAP5b were measured using an ELISA kit (Quidel**, **San Diego, CA, USA). Serum intact FGF-23 levels were measured using a two-site ELISA kit (Kainos Laboratories, Tokyo, Japan). The two specific murine monoclonal antibodies employed bind to full-length FGF-23, without inclusion of C-terminal fragments (normal range: 18 – 108 pg/mL). The kit has a sensitivity with minimum detection limit of 3 pg/mL and a quantification range from 3 to 800 pg/mL. Each sample was assayed in duplicate. When concentrations exceeded 800 pg/mL, the measurement was repeated using a 1/10 dilution. The intra- and inter-assay coefficients of variation were 4.7 and 6.4%, respectively. Serum sclerostin levels were measured using a sclerostin ELISA kit (Biomedica, Vienna, Austria). The kit has a minimum detection limit of 72.7 pg/mL. Each sample was assayed in duplicate. Samples with sclerostin measurements above upper limit of detection of the assay (5,400 pg/mL) were repeated using a 1/2 dilution. The intra- and inter-assay coefficients of variation were 8.1 and 5.5%, respectively. 

### Mineralized bone histology and bone histomorphometry 

Bone samples were 0.3 cm in diameter and at least 3 cm in length. They were fixed in ethanol at room temperature, dehydrated, and embedded in methyl methacrylate as described previously [18]. Sections were stained with the modified Masson-Goldner trichrome stain [19], the aurin tricarboxylic acid stain [20], and solochrome azurine stain [21]. Unstained sections were prepared for phase-contrast and fluorescence light microscopy. Bone histomorphometry for static and dynamic parameters of bone structure, formation, and resorption was done at a magnification × 200 using the modified OsteoMetrics system (OsteoMetrics Inc, Decatur, GA, USA). All measured histomorphometric parameters are in compliance with the recommendations of the nomenclature committee of the American Society of Bone and Mineral Research [22, 23]. Renal osteodystrophy was assessed by evaluation of its components “turnover, mineralization, and volume” (TMV) [1, 2]. Bone turnover was assessed by activation frequency (Ac.f.) (normal 0.49 – 0.72/year) and bone formation rate/bone surface (BFR/BS) (normal 1.81 – 3.80 mm^3^/cm^2^/year). Low bone turnover (LBT) was defined by Ac.f < 0.49/year and BFR/BS < 1.81 mm^3^/cm^2^/year and high bone turnover (HBT) by Ac.f > 0.72/year and BFR/BS > 3.80 mm^3^/cm^2^/year. Mineralization was assessed by osteoid thickness and mineralization lag time. Osteomalacia was defined as osteoid thickness > 20 µm combined with mineralization lag time > 100 days or osteoid maturation time > 40 days [1, 24]. Bone volume was assessed by cancellous bone volume/tissue volume and by trabecular and cortical thickness. 

### Immunohistochemistry for FGF-23 and sclerostin 

Immunohistochemical expression of FGF-23 and sclerostin in bone was determined using the established method by Pereira et al. [7]. Peroxidase activity in tissue sections was blocked by a hydrogen peroxide solution after removal of plastic. Antigen retrieval was performed with acetic acid. For blocking endogenous biotin, a biotin blocking kit (Vector Laboratories, Burlingame, CA, USA) was used. Bone sections were incubated overnight at 4 °C with affinity purified polyclonal goat anti-human FGF-23(225–244) (dilution: 1 : 500, Immutopics Intl, San Clemente, CA, USA) or affinity rabbit polyclonal anti-human sclerostin (aa 12-42, N terminal) (dilution 1 : 200, Abcam, Cambridge, MA, USA). Bone sections were incubated with biotinylated horse anti-goat or anti-rabbit secondary antibody (Vector Laboratories) followed by ABComplex/HRP complex incubation (ABC-kit, Vector Laboratories), and developed using AEC kit (Vector Laboratories). A minimum area of 10 optical fields at a magnification of × 20 of trabecular or cortical bone was assessed in each section by a single examiner blinded to histomorphometric results. Percent positivity was determined by dividing number of trabecular or cortical osteocytes with positive staining by the total number of osteocytes. 

### Statistical analyses 

Results are given as mean ± standard deviation (SD) or median and 25^th^ – 75^th^ interquartiles when values were not normally distributed. Categorical variables are expressed as percentages. Comparisons of continuous variables were done using Mann-Whitney U test. Spearman rank test was used to assess correlations. All statistical analyses were performed using SPSS version 23 (SPSS, Inc, Chicago, IL). p ≤ 0.05 was considered statistically significant. 

## Results 

108 patients aged 25 – 81 years (mean ± SD: 56 ± 13 years) were enrolled. They underwent anterior iliac crest bone biopsies after double-tetracycline labeling. Demographics and clinical and biochemical characteristics are shown in Table 1. There were 11 patients with CKD stage 2, 16 patients with CKD stage 3, 9 patients with CKD stages 4 and 5, and 64 patients with CKD-5D. Patients were on hemodialysis for 49 ± 117 months. 18 adult subjects without known kidney disease were included as controls. 

### Serum levels and bone expression of FGF-23 and sclerostin 

**Figure 1.**
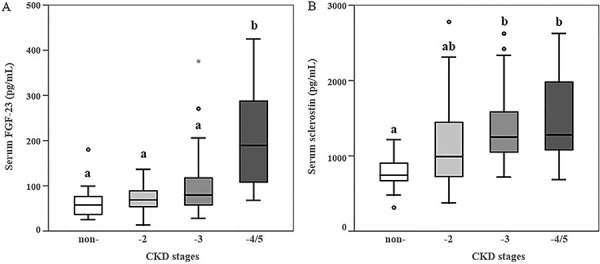
Serum levels of FGF-23 (A) and sclerostin (B) in controls and CKD stages 2 – 5D patients. Open circles represent the outliers. Median values with different letters are significantly different, p < 0.05.

**Figure 2.**
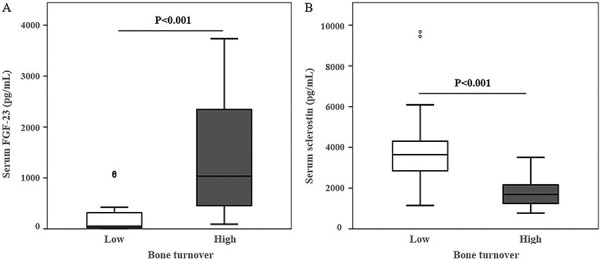
Relationship between serum levels of FGF-23 (A) and sclerostin (B) in CKD-5D patients with low- and high- bone turnover.

FGF-23 serum levels were significantly higher in patients starting at CKD-4/5 compared to lower CKD stages and to controls (Figure 1A). In patients on dialysis, FGF-23 serum levels were significantly higher in patients with high bone turnover compared to low turnover (Figure 2A). Sclerostin serum levels were significantly higher starting at CKD-3 compared to CKD-2 and controls. In CKD-5D, there was a pronounced increase in FGF-23 and sclerostin compared to lower CKD stages (Table 1). This increase was significantly higher in FGF-23 compared to sclerostin (14.7% and 2.5%, respectively). Also, in CKD-5D patients, sclerostin serum levels were higher in patients with low bone turnover than with high bone turnover (Figure 2B). 

**Figure 3.**
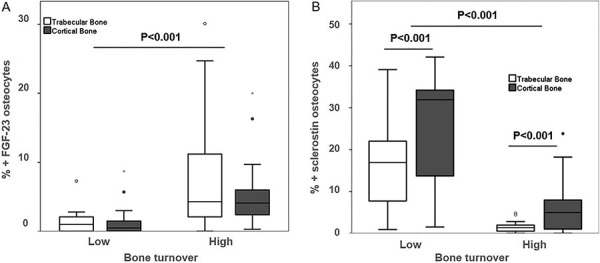
Percentage of osteocytes positive for FGF-23 (A) and sclerostin (B) per total osteocyte number of controls and CKD stages 2 – 5D patients. Values are represented by mean and standard deviation.

In bone, FGF-23 was expressed exclusively in osteocytes. It correlated positively with CKD stages (ρ = 0.55, p < 0.001) increasing from 5.3- to 7.1-fold in patients starting at CKD-2 compared to those with non-CKD. In patients with CKD-5D, FGF-23 expression was increased 18-fold. No difference in FGF-23 expression was seen between trabecular and cortical osteocytes among CKD stages (Figures 3A, Figure 5A). FGF-23 was significantly higher in CKD-5D patients with high versus low turnover. The increase in concentration of blood levels of FGF-23 (up to 3,400 mg/mL) was 50-fold in CKD-5D compared to CKD stage 2 levels, while the increase in bone osteocytes expressing FGF-23 was 18-fold. 

**Figure 4.**
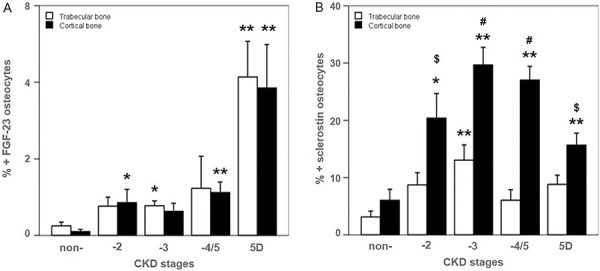
Relationship between osteocyte expression of FGF-23 (A) and sclerostin (B) and low- and high-turnover renal osteodystrophy in CKD-5D. Osteocytes positives per total osteocytes number for (A) FGF-23 (%) and for in for (B) sclerostin in bone.

**Figure 5.**
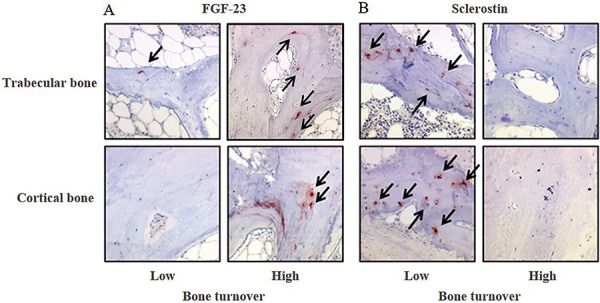
Immunohistochemistry for FGF-23 (A) and sclerostin (B) in cortical and trabecular bone with high and low bone turnover (× 200). Osteocytes positive (red nucleus) for FGF-23 (A) and for sclerostin (B) are highlighted by a black arrow. Osteocytes negative show blue nuclei.

Sclerostin was also expressed exclusively in osteocytes. It correlated positively with CKD stages (ρ = 0.63, p < 0.001) with an increase from 3.8- to 5.1-fold starting at CKD-2 compared to those with non-CKD. This increase was progressive and significantly greater in cortical than in cancellous bone (Figure 3B). In CKD-5D, its expression was significantly higher in low versus high bone turnover (Figures 4B, Figure 5B). 

### Correlations between FGF-23 and sclerostin bone expression and serum biochemical parameters 

Expressions of FGF-23 in cortical bone correlated positively with all measured serum parameters except serum calcium (Table 2). In trabecular bone, FGF-23 expression correlated only with serum levels of phosphorus and FGF-23 (Table 2). Serum FGF-23 correlated positively with serum phosphorus, BSAP, intact PTH, and sclerostin (from ρ 0.40 to 0.70) (Table 2). Higher trabecular and cortical expression of FGF-23 was associated with lower expression of sclerostin in osteocytes but it was not significant (data not shown). 

Expression of sclerostin in osteocytes of trabecular and cortical bone showed negative correlations with BSAP, intact PTH, and FGF-23 (ρ –0.37 to –0.52) (Table 2) but not with serum calcium and phosphorus, while sclerostin serum levels correlated with serum phosphorus, FGF-23, and intact PTH (ρ 0.38, 0.42 and –0.51, respectively). 

### Correlation between expression of FGF-23 and sclerostin in cortical and trabecular bone and bone histomorphometric parameters 

Expressions of FGF-23 and sclerostin in cortical and trabecular bone were strongly associated with parameters of bone turnover with positive correlations for FGF-23 and negative correlations for sclerostin. The highest correlation was found between sclerostin and osteoblast surface. Of note, there were no correlations between expression of FGF-23 and cellular bone parameters in trabecular bone. 

There was a negative correlation between expression of FGF-23 and mineralization lag time in cortical and trabecular bone; and a negative correlation between sclerostin expression in cortical and trabecular bone with osteoid thickness. Similarly, serum levels of FGF-23 and sclerostin correlated with mineralization lag time and osteoid thickness, respectively. 

FGF-23 expression was positively correlated with trabecular thickness and negatively with cortical thickness. Expression of sclerostin in cortical bone showed weak relationships with bone volume and trabecular thickness. 

## Discussion 

The obtained results confirm prior observations of increased blood levels of FGF-23 and sclerostin in patients with renal insufficiency [25, 26, 27, 28]. We also confirm prior observations of increased serum FGF-23 levels in patients with high-turnover renal osteodystrophy and better bone mineralization with very high FGF-23 levels [6, 29]. We now show expression of FGF-23 and sclerostin in osteocytes and their distribution between cortical and trabecular bone. Elevated serum levels of FGF-23 and sclerostin are seen along with higher bone expressions in renal insufficiency. This suggests that the source for the increased blood levels is at least in part bone. However, the concentration of blood levels of FGF-23 is 50-fold higher in CKD-5D compared to stage CKD-2 levels, while the increase in expression of osteocytes from CKD-2 to CKD-5D is only 18-fold. This shows that osseous production of FGF-23 only partially explains the dramatic increase in FGF-23 circulating levels, and strongly suggests an increase in extra-skeletal FGF-23 synthesis and/or osteocytic secretion as previously shown [30]. Increase of sclerostin in blood and bone from CKD-2 to CKD-5D is 3.5- and 4.9-fold and appears more parallel confirming the prevailing knowledge that sclerostin is expressed almost exclusively in bone [31]. 

Both sclerostin and FGF-23 are expressed in cortical and trabecular bone, with stronger expression of sclerostin in cortical than trabecular bone, while we did not find a difference in FGF-23 expression between trabecular and cortical osteocytes. FGF-23 expression in cortical bone correlated better than cancellous bone FGF-23 with bone turnover parameters such as activation frequency, bone formation rate, and osteoblast and osteoclast surface. This finding suggests a stronger role of cortical compared to trabecular bone in turnover activity. Sclerostin expression in osteocytes in cortical and trabecular bone was strongly and inversely associated with all dynamic and cellular turnover parameters and with osteoid thickness, whereby sclerostin expression was higher in low bone turnover. The relationship between bone turnover and sclerostin levels was stronger than the relationship between eGFR and sclerostin levels. This is in agreement with prior observations that sclerostin levels in CKD patients are mainly increased in association with abnormal bone turnover and not with reduction of eGFR [32]. 

The observed differences in sclerostin and FGF-23 expression in high versus low turnover might have to be taken into consideration when treatment modalities are designed for correction of bone turnover in CKD patients. 

Higher FGF-23 expression in cortical and trabecular bone was associated with higher trabecular thickness and volume but lower cortical thickness. Higher sclerostin expression in trabecular bone was found with higher cortical thickness, but higher sclerostin expression in cortical bone was associated with lower trabecular bone volume and lower trabecular bone thickness. The observed associations between these bone volume parameters and FGF-23 and sclerostin in bone and serum should be considered when suppression of FGF-23 and sclerostin production are treatment goals. 

The strength of this study is the availability of bone tissue for analysis of bone and serum levels of FGF-23 and sclerostin in 108 CKD patients. The limitation is its cross-sectional design, which does not allow to establish causal relationships.[Table Table3]

## Conclusion 

In conclusion, the results confirm that with decrease of kidney function we have an increase in FGF-23 and sclerostin in blood and in bone. There are differences in the site of bone expression and in the relationship between increases in bone and blood with much higher increases in FGF-23 compared to sclerostin. The increase in sclerostin appears to correlate with bone turnover while FGF-23 increases are closely related to decreased kidney function with a dramatic increase in CKD-5D. 

The observed differences in sclerostin and FGF-23 expression in high versus low turnover and differences in bone volume should be considered when treatment modalities are developed for management of high and low bone turnover abnormalities in CKD patients. Additional controlled studies with prospective design are needed. 

## Acknowledgment 

We thank Dr. Amr Mohamed for performance of bone biopsies. 

## Funding 

Research reported in this publication was supported by National Institute of Arthritis and Musculoskeletal and Skin Diseases, National Institutes of Health Award R01080770 and the Kentucky Nephrology Research Trust. The content is solely the responsibility of the authors and does not necessarily represent the official views of the National Institutes of Health. 

## Conflict of interest 

Hartmut Malluche is Editor in Chief of Clinical Nephology. All other authors have no conflict of interest. 

**Table 1.** Demographics and clinical and biochemical characteristics in patients with CKD stages 2 – 5D.

Demographic and clinical parameters	Control	CKD-2	CKD-3	CKD-4/5	CKD-5D	p-values
No. of subjects	18	11	16	9	64	
Age	55 ± 12	63 ± 10	65 ± 14	56 ± 15	53 ± 13	N.S.
Gender (F, %)	100%	73%	75%	78%	59%	
Race (Caucasian, %)	100%	100%	100%	100%	75%	
Race (Black, %)	0	0	0	0	22%	
Dialysis vintage, median (month)					66 (48 – 95)	
High turnover (%)	22%	9%	25%	22%	44%	
Patients with diabetes mellitus (%)	0	0	13%	44%	27%	
Tx with active 1,25D	11%	27%	6%	33%	40%	
Tx with phosphate binders (%)	0	0	6%	11%	97%	
Sevelamer and lanthanum (%)	0	0	0	0	48%	
Calcium containing (%)	0	0	3%	11%	62%	
Calcium (mg/dL)	9.6 ± 0.4	9.5 ± 0.5	9.2 ± 0.3	8.8 ± 1.7	9.1 ± 1.0	N.S.
Phosphorus (mg/dL)	3.6 ± 0.6^a^	3.9 ± 0.7^a^	3.6 ± 0.5^a^	4.2 ± 0.6^ab^	5.4 ± 1.6^b^	< 0.001
BSAP (U/L)	27 [22 – 32]^ab^	24 [16 – 31]^ab^	26 [20 – 29]^ab^	23 [16 – 26]^a^	38 [27 – 109]^b^	0.002
TRAP5b (U/L)	3.7 [2.6 – 4.5]^ab^	3.8 [2.9 – 4.7]^ab^	2.8 [2.1 – 3.8]^a^	2.3 [2.0 – 4.6]^ab^	5.0 [3.6 – 8.0]^b^	< 0.001
FGF-23 (pg/mL)	57 [35 – 76]^a^	67 [33 – 78]^a^	76 [62 – 89]^a^	230 [122 – 288]^a^	3,398 [458 – 11,799]^b^	< 0.001
Sclerostin (pg/mL)	744 [579 – 900]^a^	981 [834 – 1,776]^ab^	1,181 [961 – 1,249]^b^	1,205 [985 – 1,621]^b^	2,854 [1,187 – 3,727]^c^	< 0.001
1,25D (ng/mL)	58 [29 – 88]^a^	47 [25 – 57]^a^	40 [30 – 40]^ab^	42 [18 – 42]^ab^	4 [3.5 – 5.2]^b^	< 0.001
Total PTH (pg/mL)	33 [21 – 50]^a^	37 [26 – 42]^ab^	52 [31 – 77]^ab^	152 [100 – 183]^bc^	231 [68 – 645]^c^	< 0.001

Tx = treatment; BSAP = bone-specific alkaline phosphatase; TRAP5b = tartrate-resistant acid phosphatase 5b; 1,25D = 1,25-dihydroxyvitamin D; total PTH = total parathyroid hormone; FGF-23 = fibroblast growth factor-23. Results are given as mean ± SD, median [25 – 75 percentiles] or number of patients and percent (%) of total number of patients. p-value represents tests of significance from one-way ANOVA or Kruskal-Wallis. Mean or median values with different letters are significantly different, p < 0.05.

**Table 2.** Spearman’s ρ correlations between serum levels and bone expression of FGF-23 and sclerostin with serum biochemical characteristics in all CKD stages

Parameters	Serum FGF-23	% Trab FGF-23 +Ocy	% Cort FGF-23 +Ocy	Serum sclerostin	% Trab sclerostin +Ocy	% Cort sclerostin +Ocy
Calcium	0.10	–0.06	0.02	–0.03	0.10	0.09
Phosphorus	0.70**	0.40**	0.49**	0.38**	–0.15	–0.30*
BSAP	0.40**	0.09	0.35**	–0.01	–0.43**	–0.52**
Intact PTH	0.59**	0.21	0.41**	–0.15	–0.40**	–0.52**
FGF-23		0.49**	0.58**	0.42**	–0.37**	–0.50*
Sclerostin	0.42**	0.13	0.10		0.68**	0.66**

% Trab FGF-23 +Ocy = % of osteocytes positives for FGF-23 staining in trabecular bone; % Cort FGF-23 +Ocy = % of osteocytes positives for FGF-23 staining in cortical bone; % Trab sclerostin +Ocy = % of osteocytes positives for sclerostin staining in trabecular bone; % Cort sclerostin +Ocy = % of osteocytes positives for sclerostin staining in cortical bone. *p > 0.05, **p > 0.01.

**Table 3. Table3:** Spearman’s ρ correlations between serum levels and bone expression of FGF-23 and sclerostin with histomorphometric parameters in CKD-5D.

Parameters	Serum FGF-23	% Trab FGF-23 +Ocy	% Cort FGF-23 +Ocy	Serum sclerostin	% Trab Sclerostin +Ocy	% Cort Sclerostin +Ocy
Turnover						
Ac.f	0.55**	0.45**	0.60**	–0.56**	–0.69**	–0.66**
BFR/BS (mm^3^/cm^2^/y)	0.50**	0.35*	0.56**	–0.50**	–0.57**	–0.63**
Ob.S/BS (%)	0.38**	0.18	0.40**	–0.62**	–0.68**	–0.70**
Oc.S/BS (%)	**0.32** ^a^	0.15	**0.35** ^a^	–0.42**	–0.58**	–0.58**
Mineralization						
O.Th (mm)	0.10	0.03	0.23	–0.53**	–0.64**	–0.65**
Mlt (d)	–0.60**	–0.43**	–0.49**	0.04	0.02	0.08
Volume						
BV/TV (%)	0.26	0.16	0.40**	–0.32*	–0.13	**–0.29** *^a^*
Tb.Th (μm)	0.36*	0.44**	0.41**	–0.22	–0.28	–0.37*
Ct.Th (μm)	–0.55**	–0.53**	–0.55**	0.24	0.39*	0.33

% Trab FGF-23 +Ocy = % of osteocytes positives for FGF-23 staining in trabecular bone; % Cort FGF-23 +Ocy = % of osteocytes positives for FGF-23 staining in cortical bone; % Trab sclerostin +Ocy = % of osteocytes positives for sclerostin staining in trabecular bone; % Cort sclerostin +Ocy = % of osteocytes positives for sclerostin staining in cortical bone. Ac.f = activation frequency; BFR/BS = bone formation rate / bone surface; Ob.S/BS = osteoblast surface / bone surface; Oc.S/BS = osteoclast surface / bone surface; O.Th = osteoid thickness; Mlt = mineralization lag time; BV/TV = bone volume / tissue volume; Tb.Th = trabecular thickness; Ct.Th = cortical thickness. *p > 0.05, **p > 0.01.

## References

[1] Malluche HH Monier-Faugere MC Renal osteodystrophy: what’s in a name? Presentation of a clinically useful new model to interpret bone histologic findings. Clin Nephrol. 2006; 65: 235–242. 16629221 10.5414/cnp65235

[2] Moe S Drüeke T Cunningham J Goodman W Martin K Olgaard K Ott S Sprague S Lameire N Eknoyan G Definition, evaluation, and classification of renal osteodystrophy: a position statement from Kidney Disease: Improving Global Outcomes (KDIGO). Kidney Int. 2006; 69: 1945–1953. 16641930 10.1038/sj.ki.5000414

[3] Albanese L Caliendo G D’Elia G Passariello L Molinari AM Napoli C Vietri MT Diagnostic utility of FGF-23 in mineral bone disorder during chronic kidney disease. J Circ Biomark. 2022; 11: 1–4. 35023876 10.33393/jcb.2022.2328 PMC8749389

[4] Bergwitz C Jüppner H Regulation of phosphate homeostasis by PTH, vitamin D, and FGF23. Annu Rev Med. 2010; 61: 91–104. 20059333 10.1146/annurev.med.051308.111339 PMC4777331

[5] Wolf M Update on fibroblast growth factor 23 in chronic kidney disease. Kidney Int. 2012; 82: 737–747. 22622492 10.1038/ki.2012.176 PMC3434320

[6] Lima F El-Husseini A Monier-Faugere MC David V Mawad H Quarles D Malluche HH FGF-23 serum levels and bone histomorphometric results in adult patients with chronic kidney disease on dialysis. Clin Nephrol. 2014; 82: 287–295. 25208316 10.5414/CN108407 PMC4535177

[7] Pereira RC Juppner H Azucena-Serrano CE Yadin O Salusky IB Wesseling-Perry K Patterns of FGF-23, DMP1, and MEPE expression in patients with chronic kidney disease. Bone. 2009; 45: 1161–1168. 19679205 10.1016/j.bone.2009.08.008 PMC2783834

[8] Rebholz CM Grams ME Coresh J Selvin E Inker LA Levey AS Kimmel PL Vasan RS Eckfeldt JH Feldman HI Hsu CY Lutsey PL Serum fibroblast growth factor-23 is associated with incident kidney disease. J Am Soc Nephrol. 2015; 26: 192–200. 25060052 10.1681/ASN.2014020218 PMC4279743

[9] Wolf M Molnar MZ Amaral AP Czira ME Rudas A Ujszaszi A Kiss I Rosivall L Kosa J Lakatos P Kovesdy CP Mucsi I Elevated fibroblast growth factor 23 is a risk factor for kidney transplant loss and mortality. J Am Soc Nephrol. 2011; 22: 956–966. 21436289 10.1681/ASN.2010080894 PMC3083317

[10] Moldovan D Moldovan I Rusu C Kacso I Patiu IM Gherman-Caprioara M FGF-23, vascular calcification, and cardiovascular diseases in chronic hemodialysis patients. Int Urol Nephrol. 2014; 46: 121–128. 23549862 10.1007/s11255-013-0422-2

[11] Tamei N Ogawa T Ishida H Ando Y Nitta K Serum fibroblast growth factor-23 levels and progression of aortic arch calcification in non-diabetic patients on chronic hemodialysis. J Atheroscler Thromb. 2011; 18: 217–223. 21139318 10.5551/jat.5595

[12] Cejka D Herberth J Branscum AJ Fardo DW Monier-Faugere MC Diarra D Haas M Malluche HH Sclerostin and Dickkopf-1 in renal osteodystrophy. Clin J Am Soc Nephrol. 2011; 6: 877–882. 21164019 10.2215/CJN.06550810 PMC3069382

[13] Cejka D Jäger-Lansky A Kieweg H Weber M Bieglmayer C Haider DG Diarra D Patsch JM Kainberger F Bohle B Haas M Sclerostin serum levels correlate positively with bone mineral density and microarchitecture in haemodialysis patients. Nephrol Dial Transplant. 2012; 27: 226–230. 21613383 10.1093/ndt/gfr270

[14] Pelletier S Dubourg L Carlier MC Hadj-Aissa A Fouque D The relation between renal function and serum sclerostin in adult patients with CKD. Clin J Am Soc Nephrol. 2013; 8: 819–823. 23430206 10.2215/CJN.07670712 PMC3641616

[15] Delgado-Calle J Sato AY Bellido T Role and mechanism of action of sclerostin in bone. Bone. 2017; 96: 29–37. 27742498 10.1016/j.bone.2016.10.007 PMC5328835

[16] Cejka D Parada-Rodriguez D Pichler S Marculescu R Kramer I Kneissel M Gross T Reisinger A Pahr D Monier-Faugere MC Haas M Malluche HH Only minor differences in renal osteodystrophy features between wild-type and sclerostin knockout mice with chronic kidney disease. Kidney Int. 2016; 90: 828–834. 27528549 10.1016/j.kint.2016.06.019 PMC5530366

[17] Gonçalves FL Elias RM dos Reis LM Graciolli FG Zampieri FG Oliveira RB Jorgetti V Moysés RM Serum sclerostin is an independent predictor of mortality in hemodialysis patients. BMC Nephrol. 2014; 15:190. 25465028 10.1186/1471-2369-15-190 PMC4265422

[18] Malluche HH Faugere MC Atlas of mineralized bone histology. Basel, New York: Karger; 1986.

[19] Goldner J A modification of the masson trichrome technique for routine laboratory purposes. Am J Pathol. 1938; 14: 237–243. 19970387 PMC1964940

[20] Lillie PD Fullmer HM. Histopathologic Technique and Practical Histochemistry (4th ed). New York: McGraw-Hill; 1976. 534.

[21] Denton J Freemont AJ Ball J Detection and distribution of aluminium in bone. J Clin Pathol. 1984; 37: 136–142. 6198339 10.1136/jcp.37.2.136 PMC498668

[22] Dempster DW Compston JE Drezner MK Glorieux FH Kanis JA Malluche H Meunier PJ Ott SM Recker RR Parfitt AM Standardized nomenclature, symbols, and units for bone histomorphometry: a 2012 update of the report of the ASBMR Histomorphometry Nomenclature Committee. J Bone Miner Res. 2013; 28: 2–17. 23197339 10.1002/jbmr.1805 PMC3672237

[23] Parfitt AM Drezner MK Glorieux FH Kanis JA Malluche H Meunier PJ Ott SM Recker RR Bone histomorphometry: standardization of nomenclature, symbols, and units. Report of the ASBMR Histomorphometry Nomenclature Committee. J Bone Miner Res. 1987; 2: 595–610. 3455637 10.1002/jbmr.5650020617

[24] Malluche HH Mawad HW Monier-Faugere MC Renal osteodystrophy in the first decade of the new millennium: analysis of 630 bone biopsies in black and white patients. J Bone Miner Res. 2011; 26: 1368–1376. 21611975 10.1002/jbmr.309 PMC3312761

[25] Neto R Pereira L Magalhães J Quelhas-Santos J Martins S Carvalho C Frazão JM Sclerostin and DKK1 circulating levels associate with low bone turnover in patients with chronic kidney disease Stages 3 and 4. Clin Kidney J. 2021; 14: 2401–2408. 34754436 10.1093/ckj/sfab081 PMC8572981

[26] Delanaye P Cavalier E Bouquegneau A Khwaja A Sclerostin levels in CKD patients: an important, but not definitive, step on the way to clinical use. Kidney Int. 2015; 88: 1221–1223. 26649658 10.1038/ki.2015.258

[27] Kurpas A Supeł K Idzikowska K Zielińska M FGF23: A Review of Its Role in Mineral Metabolism and Renal and Cardiovascular Disease. Dis Markers. 2021; 2021: 8821292. 34055103 10.1155/2021/8821292 PMC8149241

[28] Courbebaisse M Lanske B Biology of Fibroblast Growth Factor 23: From Physiology to Pathology. Cold Spring Harb Perspect Med. 2018; 8: 8. 10.1101/cshperspect.a031260 PMC5932574 28778965

[29] Wesseling-Perry K Pereira RC Wang H Elashoff RM Sahney S Gales B Jüppner H Salusky IB Relationship between plasma fibroblast growth factor-23 concentration and bone mineralization in children with renal failure on peritoneal dialysis. J Clin Endocrinol Metab. 2009; 94: 511–517. 19050056 10.1210/jc.2008-0326 PMC2646517

[30] Courbon G Spindler JJ Von Drasek JC Martin A David V Paracrine FGF23 Signaling Suppresses Erythropoiesis in Iron Deficiency Anemia (abstract). ASN Kidney Week, November 3, 2022, Orlando, FL, USA.

[31] Robling AG Niziolek PJ Baldridge LA Condon KW Allen MR Alam I Mantila SM Gluhak-Heinrich J Bellido TM Harris SE Turner CH Mechanical stimulation of bone in vivo reduces osteocyte expression of Sost/sclerostin. J Biol Chem. 2008; 283: 5866–5875. 18089564 10.1074/jbc.M705092200

[32] Tartaglione L Pasquali M Rotondi S Muci ML Leonangeli C Farcomeni A Fassino V Mazzaferro S Interactions of sclerostin with FGF23, soluble klotho and vitamin D in renal transplantation. PLoS One. 2017; 12: e0178637. 28558021 10.1371/journal.pone.0178637 PMC5448809

